# Functional role of voltage gated Ca^2+^ channels in heart automaticity

**DOI:** 10.3389/fphys.2015.00019

**Published:** 2015-02-02

**Authors:** Pietro Mesirca, Angelo G. Torrente, Matteo E. Mangoni

**Affiliations:** ^1^Laboratory of Excellence in Ion Channel Science and Therapeutics, Département de Physiologie, Institut de Génomique FonctionnelleMontpellier, France; ^2^UMR-5203, Centre National de la Recherche Scientifique, Universités de Montpellier 1 and 2Montpellier, France; ^3^INSERM U 1191, Département de Physiologie, Universités de Montpellier 1 and 2Montpellier, France

**Keywords:** heart automaticity, L-type Ca^2+^ channel, T-type Ca^2+^ channels, sinoatrial node, atrioventricular node

## Abstract

Pacemaker activity of automatic cardiac myocytes controls the heartbeat in everyday life. Cardiac automaticity is under the control of several neurotransmitters and hormones and is constantly regulated by the autonomic nervous system to match the physiological needs of the organism. Several classes of ion channels and proteins involved in intracellular Ca^2+^ dynamics contribute to pacemaker activity. The functional role of voltage-gated calcium channels (VGCCs) in heart automaticity and impulse conduction has been matter of debate for 30 years. However, growing evidence shows that VGCCs are important regulators of the pacemaker mechanisms and play also a major role in atrio-ventricular impulse conduction. Incidentally, studies performed in genetically modified mice lacking L-type Ca_v_1.3 (Ca_v_1.3^−/−^) or T-type Ca_v_3.1 (Ca_v_3.1^−/−^) channels show that genetic inactivation of these channels strongly impacts pacemaking. In cardiac pacemaker cells, VGCCs activate at negative voltages at the beginning of the diastolic depolarization and importantly contribute to this phase by supplying inward current. Loss-of-function of these channels also impairs atrio-ventricular conduction. Furthermore, inactivation of Ca_v_1.3 channels promotes also atrial fibrillation and flutter in knockout mice suggesting that these channels can play a role in stabilizing atrial rhythm. Genomic analysis demonstrated that Ca_v_1.3 and Ca_v_3.1 channels are widely expressed in pacemaker tissue of mice, rabbits and humans. Importantly, human diseases of pacemaker activity such as congenital bradycardia and heart block have been attributed to loss-of-function of Ca_v_1.3 and Ca_v_3.1 channels. In this article, we will review the current knowledge on the role of VGCCs in the generation and regulation of heart rate and rhythm. We will discuss also how loss of Ca^2+^ entry through VGCCs could influence intracellular Ca^2+^ handling and promote atrial arrhythmias.

## Introduction

Pacemaker activity in the heart is generated by specialized myocytes, able to generate periodical oscillations of their membrane potential. These cells are thus called “pacemaker” cells (Mangoni and Nargeot, 2008). Pacemaker cells are localized in the sino-atrial node (SAN), which is a thin tissue located in the right atrium (for anatomical description see Dobrzynski et al., 2005). Under physiological conditions the cardiac impulse has origin in the SAN. The pacemaker impulse spreads from the SAN to the cardiac conduction system (composed by the atrioventricular node and Purkinje fibers network), driving the contraction of the whole working myocardium. In comparison to the rest of the conduction system, the SAN generates the fastest intrinsic automaticity, thereby inhibiting pacemaking in the atrioventricular node (AVN) and the Purkinje fibers network. Nevertheless, in case of SAN failure, the AVN can take over as dominant pacemaker center. Under conditions of atrioventricular block, Purkinje fibers are able to generate viable rhythm, even if at relatively low rates (James, 2003; Dobrzynski et al., 2013).

The generation of the automaticity in cardiac pacemaker cells is due to the diastolic depolarization, a spontaneous slowly depolarizing phase of the action potential cycle. During this phase the membrane potential progressively becomes less negative until it reaches the threshold for triggering a new action potential. The SAN action potential cycle length determines the heart rate. At the level of the individual SAN cell, different classes of ion channels of the plasma membrane, the sarcoplasmic reticulum (SR) and mytochondria contribute to the generation and regulation of automaticity, but their respective functional roles and interactions are still not fully understood.

In the recent past, two distinct, but not mutually exclusively, hypotheses were proposed to explain the mechanism underling the cardiac automaticity: the so-called “membrane clock” model of pacemaking, which considers the “funny” current (*I_f_*), an inward Na^+^/K^+^ current activated by membrane hyperpolarization at negative voltages (Brown et al., 1979) and regulated directly by cAMP (Difrancesco and Tortora, 1991) as the key initiator of the diastolic depolarization (Difrancesco, 1991). In the “calcium clock” model of pacemaking the key mechanism in the diastolic depolarization is a spontaneous rhythmic phenomenon of Ca^2+^ release from the SR activating the Na^+^/Ca^2+^ exchanger (NCX) in forward mode. This NCX mediated inward current is able to depolarize the membrane voltage to the threshold of the following action potential (Bogdanov et al., 2001; Vinogradova et al., 2002). Recently, the Ca^2+^ clock view of pacemaking has been updated into the “coupled-clock” model (Lakatta et al., 2010). In the coupled-clock model of pacemaking, the activity of membrane ion channels and spontaneous Ca^2+^ release mutually entrain to generate pacemaking (Lakatta et al., 2010; Monfredi et al., 2013). For a more extended description of the issues raised by the *I_f_* - based and the Ca^2+^ or coupled-clock models of pacemaking, the reader is referred to recent review by the principal authors (Difrancesco, 2010; Lakatta et al., 2010; Monfredi et al., 2013).

However, either the *I_f_* -based or the coupled clock models of pacemaking do not fully appreciate the role of VGCCs in pacemaking. Indeed, in the *I_f_* -based model of pacemaking the L-type Ca^2+^ current (*I_Ca,L_*) is considered only as a determinant of the action potential upstroke and duration (Difrancesco, 1993, 2010). In the coupled-clock model of pacemaking, *I_Ca,L_* is considered as a major mechanism to replenish SR Ca^2+^ content at each pacemaker cycle (Vinogradova et al., 2002). Finally, both the *I_f_* -based and the coupled-clock models grant only a limited role to T-type VGCCs (Vinogradova et al., 2002).

However, during the last 10 years evidence accumulated showing that VGCCs contribute directly to pacemaking by carrying inward current during the diastolic depolarization phase (Zhang et al., 2002, 2011; Mangoni et al., 2003, 2006b; Marger et al., 2011a) or by stimulating the NCX activated by subsarcolemmal Ca^2+^ release during the diastolic depolarization (Lakatta et al., 2010). VGCCs also participate to the upstroke phase of the action potential (Hagiwara et al., 1988; Doerr et al., 1989; Marger et al., 2011a). Here we will focus on two distinct families of VGCCs, the L-type and the T-type Ca^2+^ channels. L-type VGCCs are expressed throughout the myocardium and are sensitive to antagonist and agonist dihydropyridines (DHPs) such as nifedipine and BAY K 8644 and are stimulated by PKA-dependent phosporylation (Striessnig, 1999; van der Heyden et al., 2005). In comparison with T-type channels, L-type VGCCs activate upon membrane depolarization at more positive potential, have Ca^2+^ and voltage dependent inactivation, as well as a higher single channel conductance (Perez-Reyes, 2003). T-type VGCCs are activated at more negative potentials than L-type VGCCs. The kinetic hallmark of native and heterogously expressed T-type mediated Ca^2+^ current is slow criss-crossing activation and fast voltage dependent inactivation (Carbone and Lux, 1987). Table 1 summarizes the main characteristics of the L- and T-type VGCCs isoforms involved in cardiac automaticity. The elucidation of the functional role of the cardiac VGCCs can give important insights into the mechanisms underlying different SAN and conduction system pathologies. Indeed, failure of generating the cardiac impulse underlies SAN bradycardia and rhythmic disease. Diseases of the sinus node account for more than 1,000,000 electronic pacemaker implantations each year. SAN disease is characterized by various symptoms including severe sinus bradycardia, sinus pauses or arrest, chronotropic incompetence, sinus node exit block (Dobrzynski et al., 2007). Heart failure, cardiomyopathy, administration of antiarrhythmic drugs and other acquired cardiac conditions can induce SAN dysfunction. Nevertheless, in a significant number of patients, SAN dysfunction shows inherited features (Sarachek and Leonard, 1972; Lehmann and Klein, 1978; Mackintosh and Chamberlain, 1979; Dobrzynski et al., 2007; Sanders et al., 2014). Mutations in genes regulating L-type VDCCs involved in SAN automaticity such as L-type Ca_v_1.3 (Mangoni et al., 2003; Baig et al., 2011) and T-type Ca_v_3.1 (Marger et al., 2011a; Strandberg et al., 2013) are associated with various forms of previously unexplained tachy-brady syndromes and conduction defects (Mangoni and Nargeot, 2008; Pfeufer et al., 2010).

**Table 1:** **Characteristics of the L- and T-type VGCCs isoforms involved in cardiac automaticity**.

	**L-type VGCC (Ca_v_1)**		**T-type VGCC (Ca_v_3)**	
	**Ca_v_1.2**	**Ca_v_1.3**	**Ca_v_3.1**	**Ca_v_3.2**
Expression time	Embryonic stage	Embryonic stage	Start to increase in the perinatal period and becomes predominant in the adulthood	High in Embryonic heart tissue and then decrease and disappear in adult heart
Cardiac tissues expression	SAN, AVN, atria, PF networks, Ventricles	SAN, AVN, atria, PF networks, poorly or not expressed in ventricular	SAN, AVN, atria, PF networks, poorly or not expressed in ventricular tissue	SAN, AVN, atria, PF networks, poorly expressed in ventricular tissue
Voltage dependent activation	High threshold of activation (~−40 mV) Fast activation	Lower threshold of activation than Ca_v_1.2 (~−55 mV) Fast activation	Lower threshold of activation (~−70 mV) Slow activation	
Inactivation properties	Ca^2+^ and voltage dependent inactivation	Ca^2+^ and voltage dependent inactivation	Fast voltage dependent inactivation	
DHP sensitivity	High	Lower than Ca_v_1.2	Low and very low	
Role in pacemaking	Control the Ca^2+^ dependent upstroke phase of action potential	Diastolic pacemaker current	Diastolic pacemaker current	
Knock-out mice phenotype	Lethal	Strong bradycardia, SAN arrhythmia, conduction system dysfunction	Mild bradycardia AV conduction disorders	No phenotype

## Cardiac voltage gated Ca^2+^ channels: molecular determinants and expression

VGCCs are an important pathway for Ca^2+^ entry in pacemaker cells. In the mammalian heart, L- and T-type mediated Ca^2+^ currents are expressed in SAN, AVN, and Purkinje Fibers network and they have been consistently recorded in pacemaker SAN and AVN cells (Tseng and Boyden, 1989; Mangoni et al., 2003, 2006b; Marger et al., 2011a). Hagiwara et al. (1988) were the first to report the expression of *I_Ca,L_* in isolated SAN pacemaker cells and to describe its kinetic and pharmacologic properties. In particular, they defined *I_Ca,L_* as a “high”-threshold Ca^2+^ current activated from about -30 mV and distinguished from T-type mediated Ca^2+^ current (*I_Ca,T_*), a “low” threshold Ca^2+^ current activated at −50 mV, suggesting that both currents participate the latter half of the slow diastolic depolarization (Hagiwara et al., 1988).

L-type VGCCs are hetero-oligomeric complexes constituted by a voltage sensitive pore, the so–called α1-subunits, together with different accessory subunits (α2δ, β, and γ) (Striessnig, 1999) and they are highly sensitive to DHP Ca^2+^ channels modulators. Four α1-subunits have been cloned and classified for the L- type Ca^2+^ channel, namely Ca_v_1.1, Ca_v_1.2 Ca_v_1.3, and Ca_v_1.4 (Catterall et al., 2003). Ca_v_1.1 subunits are expressed in the skeletal muscle, where they couple membrane excitation to contraction (Tanabe et al., 1988; Tuluc et al., 2009), Ca_v_1.4 expression is predominant in the retina, spinal cord and immune cells (McRory et al., 2004; Striessnig and Koschak, 2008). Ca_v_1.2 and Ca_v_1.3 are expressed in neurons, as well as in cells from the neuroendocrine and cardiovascular systems (Catterall, 2000). Ca_v_1.2 is expressed in the whole heart but predominantly in atria and ventricles; Ca_v_1.3 expression is predominant in the supraventricular regions with higher amounts of Ca_v_1.3 in the rhythmogenic centers (Marionneau et al., 2005). Electrophysiological measurements showed clear differences between Ca_v_1.3 and Ca_v_1.2 mediated *I_Ca,L_*. Ca_v_1.3-mediated *I_Ca,L_* activates at more negative voltages and displays slower current inactivation during depolarization allowing these channels to mediate long lasting Ca^2+^ influx during weak depolarization (Platzer et al., 2000; Koschak et al., 2001). As showed by Hagiwara (Hagiwara et al., 1988), T-type VGCCs are activated at more negative potentials than L-type VGCCs. Moreover, *I_Ca,T_* have faster voltage-dependent inactivation and inactivation is complete at more negative membrane potentials than *I_Ca,L_* (Perez-Reyes, 2003) (Table 1).

Three genes encoding for T-type α-subunits have been cloned and named Ca_v_3.1, Ca_v_3.2, and Ca_v_3.3. While the Ca_v_3.3 isoform is not present in the heart, the expression of Ca_v_3.1 and Ca_v_3.2 isoforms in the myocardium varies according to the developmental status of the tissue. Ca_v_3.2 constitutes the predominant T-type isoform in embryonic heart tissue (Ferron et al., 2002); Ca_v_3.1 channels expression increases during perinatal period and reaches its maximal in adulthood (Marshall et al., 1993). In adult SAN Ca_v_3.1 expression is higher than Ca_v_3.2 (Bohn et al., 2000). Contrary to the Ca_v_1 family, the Ca_v_3 family is almost insensible to DHPs and at present, no selective inhibitor to discriminate the contribution of Ca_v_3.1 and Ca_v_3.2 channels to the total *I_Ca,T_* is available.

## Regulation of L- and T-type Ca^2+^ channels in cardiac tissues

Cardiac VGCCs are subject of multiple regulatory mechanisms involving both intramolecular regulatory sites and interactions with cellular second messengers and kinases.

Ca^2+^ influx through VGCCs can “auto-regulate” the channel activity in a negative (CDI, Ca^2+^-dependent inactivation) or positive (CDF, Ca^2+^-dependent facilitation) manner. L-type Ca^2+^ channels undergo calmoduline-mediated CDI or calmoduline kinase II (CaMKII)-mediated CDF (Christel and Lee, 2012). On the contrary only CaMKII-mediated CDF has been described for T-type Ca^2+^ channels (Christel and Lee, 2012).

It has been shown that SAN L-type Ca^2+^ channels undergo voltage-dependent inactivation (VDI) and facilitation (VDF) (Mangoni et al., 2000; Christel et al., 2012). Christel et al. (2012) showed that Ca_v_1.2-mediated *I_Ca,L_* undergoes stronger VDI than Ca_v_1.3-mediated *I_Ca,L_* and that Ca_v_1.3-mediated *I_Ca,L_* exhibited stronger VDF than Ca_v_1.2 -mediated *I_Ca,L_*. Numerical modeling simulations predicted that VDF was responsible of 25% increase in Ca_v_1.3-mediated *I_Ca,L_* which, as a consequence, induced a small positive chronotropic effect. These data further support the importance of Ca_v_1.3 Ca^2+^ channels regulation in cardiac pacemaker activity.

L-type Ca^2+^ channels are also potently regulated by cAMP-dependent protein kinase A (De Jongh et al., 1996; Ramadan et al., 2009). Regulation of T-type Ca^2+^ channels by cAMP dependent protein kinase A is still controversial (Chemin et al., 2006), however, in a recent work Li et al. (2012) found that in cardiac myocytes the activity of Ca_v_3.1 T-type VGCCs was significantly increased by isoproterenol, a β-adrenergic agonist, and that this regulation was strictly connected to the adenylate cyclase/cAMP/PKA machinery similar to L-Type Ca^2+^ channels. One of the most important differences in the pharmacologic modulation of T- and L-type Ca^2+^ channels rises from their different sensitivity to DHPs. DHPs are known to act on *I_Ca,L_* without affecting *I_Ca,T_* (Hagiwara et al., 1988). Nevertheless, this concept has been challenged by different studies showing an effect of certain types of DHPs also on different subunits of T-type VGCCs (Bladen et al., 2014a,b). L-type Ca^2+^ channel voltage-dependence and expression are potently regulated by β subunits (see Buraei and Yang, 2010, for recent review). In the SAN, the predominant β subunit isoform expressed appears to be β2 (Marionneau et al., 2005). Co-expression of the β2 subunit with the Ca_v_1.2 α1 subunit induces slowing of the voltage dependent inactivation of *I_Ca,L_* (Cens et al., 1996). It has been proposed that β subunits regulate α1 protein trafficking (Buraei and Yang, 2013). It will be interesting to investigate whether β2 subunits regulates L-type Ca^2+^ channel trafficking in the cardiac conduction system. It has been showed that L-type Ca^2+^ channels, even to a lesser extent than other VGCCs such as T-type Ca^2+^ channels, are regulated also by phospholipids (Suh and Hille, 2005).

## Functional role of L-type and T-type Ca^2+^ channels in cardiac automaticity

### L-type Ca^2+^ channels

Evidence for the importance of L-type Ca^2+^ current in SAN pacemaking have been reported by different studies (see Mangoni et al., 2006a, for review). Kodama et al. (1997) showed that blocking *I_Ca,L_* by 2 μM nifedipine abolished the action potential in the primary central pacemaker area in rabbit SAN but not in spontaneously beating tissue from the periphery of the SAN. In contrast, they showed that tetrodotoxin 20 μM had no effect on electrical activity in the primary central pacemaker area, but depolarized the takeoff potential, decreased the upstroke velocity and slowed the spontaneous activity in nodal tissue from SAN periphery. These results are in line with the view that the rabbit pacemaker action potential strongly depends from *I_Ca,L_* in the central area of SAN but not in the periphery, where it is more sensitive to Na^+^ current (*I_Na_*). The heterogeneity of sensitivity to *I_Ca,L_* in pacemaker cells highlights the problem of isolating the contribution of *I_Ca,L_* to the diastolic depolarization phase from its contribution to the upstroke phase of the cardiac action potential.

Doerr et al. (1989) tried to overcome this major problem using the action potential clamp technique to evaluate the contribution of *I_Ca,L_* in the pacemaker cycles in rabbit isolated SAN cells. They reported a net methoxyverapamil (L-type Ca^2+^ channels blocker)-sensitive current measurable during the early diastolic depolarization as well a long lasting component during the plateau phase. Verheijck et al. (1999) have recorded the net nifedipine-sensitive *I_Ca,L_* at different times during action potential cycle. Notably, they provided direct evidence that *I_Ca,L_* can be activated at potential as negative as −60 mV, typical of the early diastolic depolarization phase, and then increases up to the threshold potential supplying inward current during the entire diastolic depolarization. In comparison to the previous study by Hagiwara et al. (1988), Verheijck and co-workers recorded *I_Ca,L_* starting from negative holding potentials (−90 mV), preventing partial steady-state inactivation of *I_Ca,L_* at negative voltages. Second, Verheijck and co-workers employed a recording protocol combining current clamp, to let the cell to depolarize and repolarize spontaneously, with voltage clamping at discrete voltages spanning the diastolic depolarization to record *I_Ca,L_*. This strategy allowed accurate measurement of the *I_Ca,L_* current density in this phase.

Moreover, they demonstrated the presence of a “low-voltage”-activated *I_Ca,L_* component, pharmacologically distinct from *I_Ca,T_*, in the diastolic depolarization range, opening the way to the description of the functional role of Ca_v_1.3 L-type channels in pacemaking.

**Figure 1:**
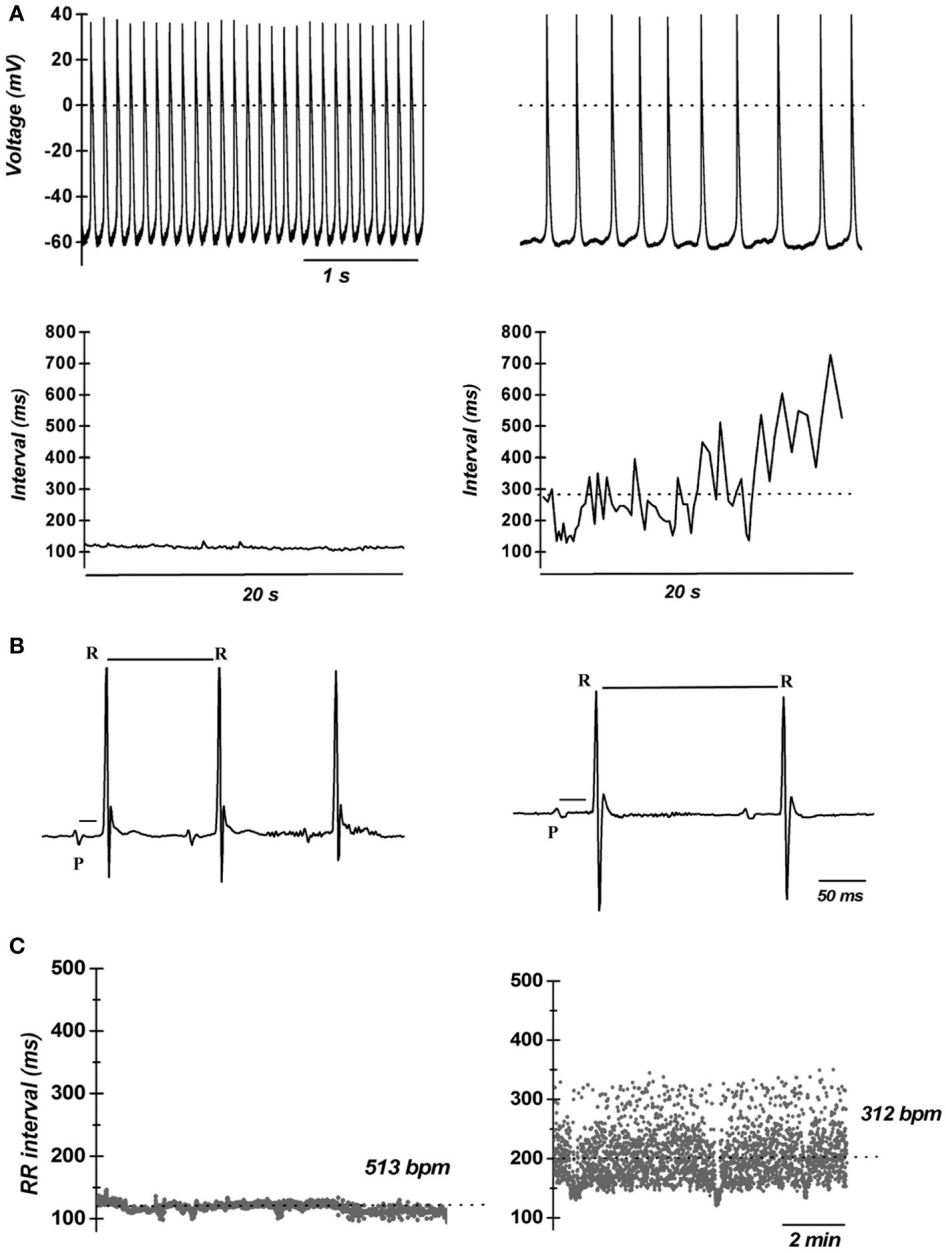
**Importance of L-type VGCCs in cardiac automaticity**. **(A)** Representative recordings of consecutive action potentials recorded in pacemaker cells from wild-type (top left panel) and Ca_v_1.3^−/−^ mice (top right panel). Cellular arrhythmia is evident as irregular cycle length duration in Ca_v_1.3^−/−^ cells (bottom right panel) compared with wild-type cells (bottom left panel). Dotted lines indicate the zero voltage level (Data from Mangoni et al., 2003). **(B)** Telemetric ECGs showing prolongation of RR interval PQ interval in Ca_v_1.3^−/−^ mice (top right panel) with respect to wild type littermates (top left panel). **(C)** Dot plot of beat to beat variability in wild-type (left panel) and Ca_v_1.3^−/−^ mice (right panel) observed during 10 min recordings. Note the dispersion of the RR intervals in Ca_v_1.3 knockout mice, revealing strong sinus arrhythmia. The dotted lines indicate the average heart rate as the number of beats per minutes (bpm) (reprinted from Mangoni et al., 2006a, with permission from Elsevier).

**Figure 2:**
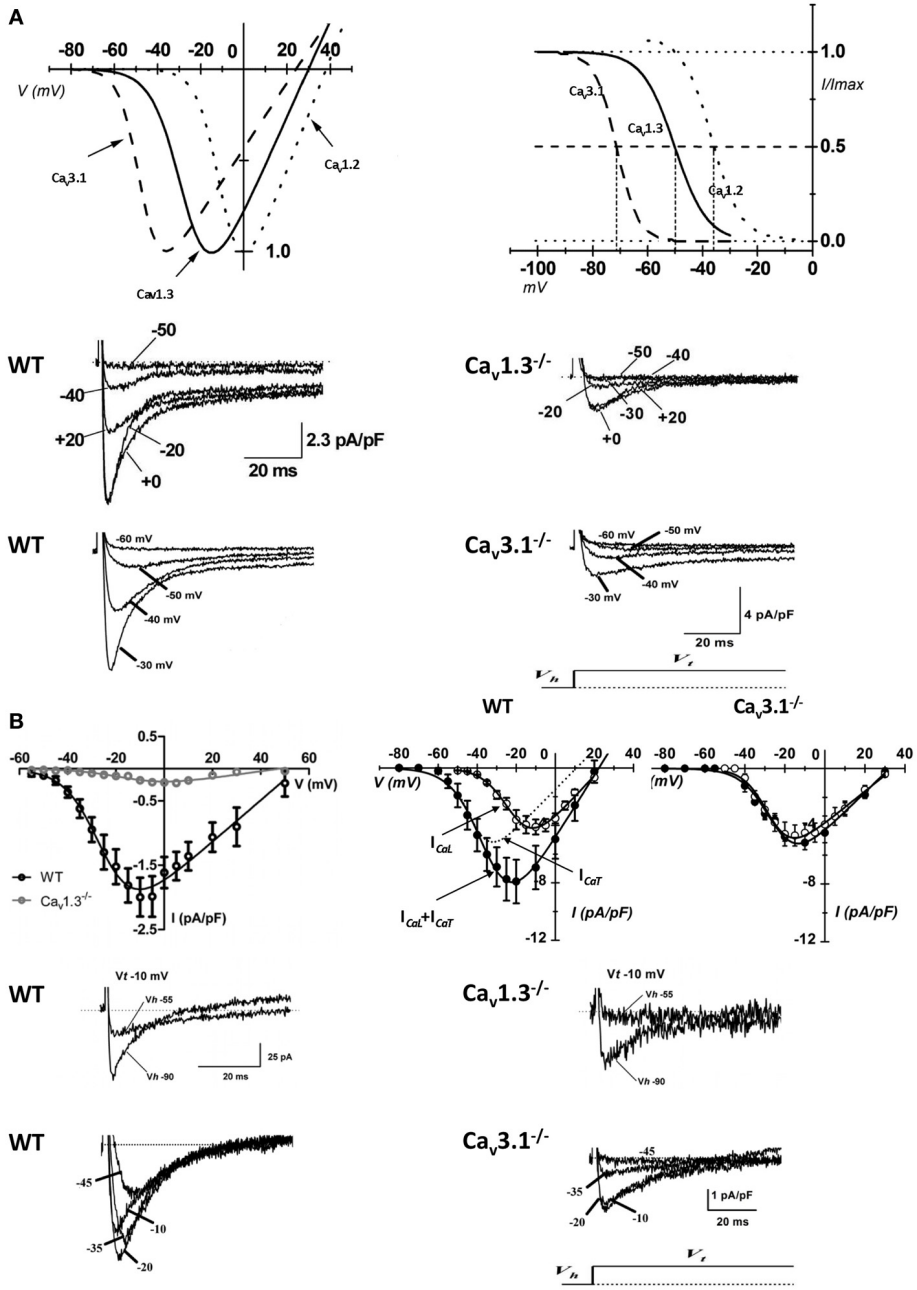
**Properties of VGCCs in cardiac pacemaker cells**. **(A)** I–V curve (top left panel) and steady-state inactivation (top right panel) of native SAN Ca_v_3.1 (dashed curve), Ca_v_1.3 (solid curve), and Ca_v_1.2 (dotted curve) channels (reprinted from Mangoni et al., 2006a, with permission from Elsevier). Examples of voltage dependent calcium currents recorded in pacemakers cells from WT (middle and bottom left panel), Ca_v_1.3^−/−^ (middle right panel) and Ca_v_3.1^−/−^ mice (bottom right panel). **(B)** Top left panel: I–V curve of L-type Ca^2+^ channels obtained from WT (black open circles) and Ca_v_1.3^−/−^ (gray open circles) isolated AVN cells. Top right panels: current to voltage relationship in isolated AVN cells from WT and Ca_v_3.1^−/−^ mice. Sample traces of *I_Ca,L_* (middle panels) and *I_Ca,T_* (bottom panels) recorded in isolated AVN cells from WT, Ca_v_1.3^−/−^ and Ca_v_3.1^−/−^ mice. For *I_Ca,L_* recordings the holding potential (V_h_) was set at −55 mV, for Ca_v_3.1 at −90 mV. Test potential (V_t_) is reported near the trace (reprinted from Mangoni et al., 2006b with permission from Wolters Kluwer Health).

The first *in vivo* evidence of the contribution of *I_Ca,L_* in cardiac pacemaker was provided by Lande et al. (2001); they recorded DHP-induced bradycardia in anesthetized mice. Subsequently, the unexpected result that electrocardiogram (ECG) recordings revealed SAN dysfunction (bradycardia and arrhythmia) in mice lacking L-type Ca_v_1.3 channels was the first genetic evidence of their importance in heart automaticity (Platzer et al., 2000). Two independent studies showed that Ca_v_1.3 channels have a key role in automaticity both *in vitro* (Figure 1A) and *in vivo* (Figures 1B,C) (Zhang et al., 2002; Mangoni et al., 2003) also unmasking important differences between Ca_v_1.3-mediated and Ca_v_1.2-mediated *I_Ca,L_* (Figure 2A). The heart chambers histology and thickness as well SAN and AVN structure did not show any differences between Ca_v_1.3^−/−^ and the wild type mice, suggesting that inactivation of Ca_v_1.3 channels has no effect on heart structure (Matthes et al., 2004). Inactivation of Ca_v_1.3-mediated *I_Ca,L_* impairs pacemaking and atrioventricular conduction, but has no effect on myocardial contractility (Matthes et al., 2004). Zhang et al. (2005) showed that intracardiac atrial stimulation induced atrial fibrillation and atrial flutter in Ca_v_1.3^−/−^ mice but not in wild-type littermates even in the absence of vagal stimulation with carbachol, a muscarinic agonist which is known to induce atrial fibrillation in control mice (Kovoor et al., 2001). In contrast, no ventricular arrhythmias were induced in either the wild-type or mutant mice (Zhang et al., 2005). These data further support the view of an important functional role of Ca_v_1.3 in the atria.

Using a knock-in mouse strain in which the DHP sensitivity in Ca_v_1.2 α1 subunits was eliminated (Ca_v_ 1.2^DHP−/−^), without affecting channel function and expression, it has been possible to separate the DHP effects of Ca_v_1.2 from those of Ca_v_1.3 and other L-Type Ca^2+^ channels.

The heart rate reducing effect induced by DHP (isradipine) in Ca_v_1.2^DHP−/−^ mice demonstrated that Ca_v_1.3 is the major L-Type Ca^2+^ channel controlling diastolic depolarization (Sinnegger-Brauns et al., 2004).

Recently, Christel et al. (2012) showed a differential degree of co-localization between the ryanodine receptors (RYRs) of the SR and Ca_v_1.3 or Ca_v_1.2 channels in primary SAN pacemaker cells. The strong co-localization of Ca_v_1.3 with RYR2 may be relevant for the functional role of RYR-mediated Ca^2+^ release in pacemaking (Vinogradova et al., 2002). During the late phase of the diastolic depolarization, RYR-mediated Ca^2+^ release promotes NCX activation, which accelerates reaching the threshold of the SAN action potential upstroke (Vinogradova et al., 2002). Close apposition of Ca_v_1.3 with RYRs may facilitate SR Ca^2+^ release since *I_Ca,L_* stimulates RYR open probability. In this respect, numerical simulations predicted that the slope of rise of diastolic RYR-dependent Ca^2+^ release increased as a function of Ca_v_1.3-mediated *I_Ca,L_* half-activation voltage (Christel et al., 2012). The coupling of this SR Ca^2+^ release to the depolarizing influence of NCX should accelerate attainment of the threshold for action potential firing of SAN cells (Vinogradova et al., 2002). L-type Ca^2+^ channels, and in particular Ca_v_1.3 channels, have been shown to physically associate with RYR2 in the nervous system (Ouardouz et al., 2003; Kim et al., 2007). It will be interesting to investigate whether such a coupling also exists in SAN cells. As previously mentioned, Ca_v_1.3^−/−^ mice show slowing of atrioventricular conduction suggesting that these channels are important in AVN physiology (Figure 2B). It has been shown that Ca_v_1.3 channels play a key role in pacemaking of AVN cells (Marger et al., 2011a; Zhang et al., 2011). In Ca_v_1.3^−/−^ AVN cells pacemaker activity is stopped and exhibited a depolarized membrane potential of -30 mV (Marger et al., 2011a) likely due the loss of crosstalk between Ca_v_1.3 channels and SK2 K^+^ channels. Indeed, functional coupling between Ca_v_1.3 and SK channels has been reported in mouse atrial myocytes (Lu et al., 2007), where Ca_v_1.3 loss-of-function prolongs the action potential duration via reduction in the activity of SK channels. Interestingly, Zhang et al. (2008) showed that mice lacking SK2 channels exhibited significant sinus bradycardia with prolongation of the atrioventricular conduction intervals (PQ intervals), thus revealing a function role of these channels in AVN automaticity.

Saturating doses of the non-selective β-adrenergic agonist isoproterenol did not restore pacemaking in Ca_v_1.3^−/−^ AVN cells. Cellular automaticity could be initiated by injection of hyperpolarizing current to drive the membrane voltage to the maximum diastolic potential of −60 mV recorded in wild-type AVN cells. When this maximum diastolic potential voltage is maintained by constant hyperpolarizing current injection, AVN cells were able to fire spontaneous action potentials. However, this firing was very slow and arrhythmic. Furthermore, the slope of the diastolic depolarization in current injected Ca_v_1.3^−/−^ cells was very low. Indeed, during the diastolic phase, only sub-threshold low amplitude oscillations of the membrane potential were recorded. These oscillations occasionally met the threshold to evoke an action potential (Marger et al., 2011a). These data indicated that Ca_v_1.3 channels have a key role in the generation of the diastolic depolarization in AVN pacemaker cells (Marger et al., 2011a). β-adrenergic stimulation induced by isoproterenol was able to increase the firing rate in current-injected Ca_v_1.3^−/−^ AVN cells. However, the firing rate of isoproterenol treated Ca_v_1.3^−/−^ myocytes did not reach the value of control AVN cells (Marger et al., 2011a).

The lack of spontaneous automaticity in Ca_v_1.3^−/−^ AVN cells *in vitro* does not imply un-excitability *in vivo*. A potential explication is that Ca_v_1.3^−/−^ cells embedded in tissue are kept at hyperpolarized membrane voltages by the electrotonic influence of the atrium (Verheijck et al., 2002), allowing the discharge of the *I_Na_* dependent action potential in the presence of SAN impulse (Marger et al., 2011a). Incidentally, Marger et al. (2011b) showed that *I_Na_* has an important role in the AVN automaticity as 20 μM TTX completely stop firing in AVN cells. Finally, it is well known that AVN is composed by different cell types, automatic and non-automatic, interacting each other and eventually implicated in different conduction pathways. These aspects too can explain the reason why Ca_v_1.3^−/−^ mice do not show complete atrioventricular block.

### T-type calcium channels

*I_Ca,T_* has been consistently found in all the three rhytmogenic centers of the heart: the SAN (Hagiwara et al., 1988; Fermini and Nathan, 1991), the AVN (Liu et al., 1993) and Purkinje fibers (Hirano et al., 1989; Tseng and Boyden, 1989) suggesting that T-type VGCCs may constitute a relevant mechanism in the generation of the diastolic depolarization.

Genetically modified mice with target inactivation of Ca_v_3.2 and Ca_v_3.1 subunit importantly helped to elucidate the role of T-type channel isoforms in cardiac pacemaking and impulse conduction (Figure 2A) (Chen et al., 2003; Mangoni et al., 2006b; Thuesen et al., 2014).

**Figure 3:**
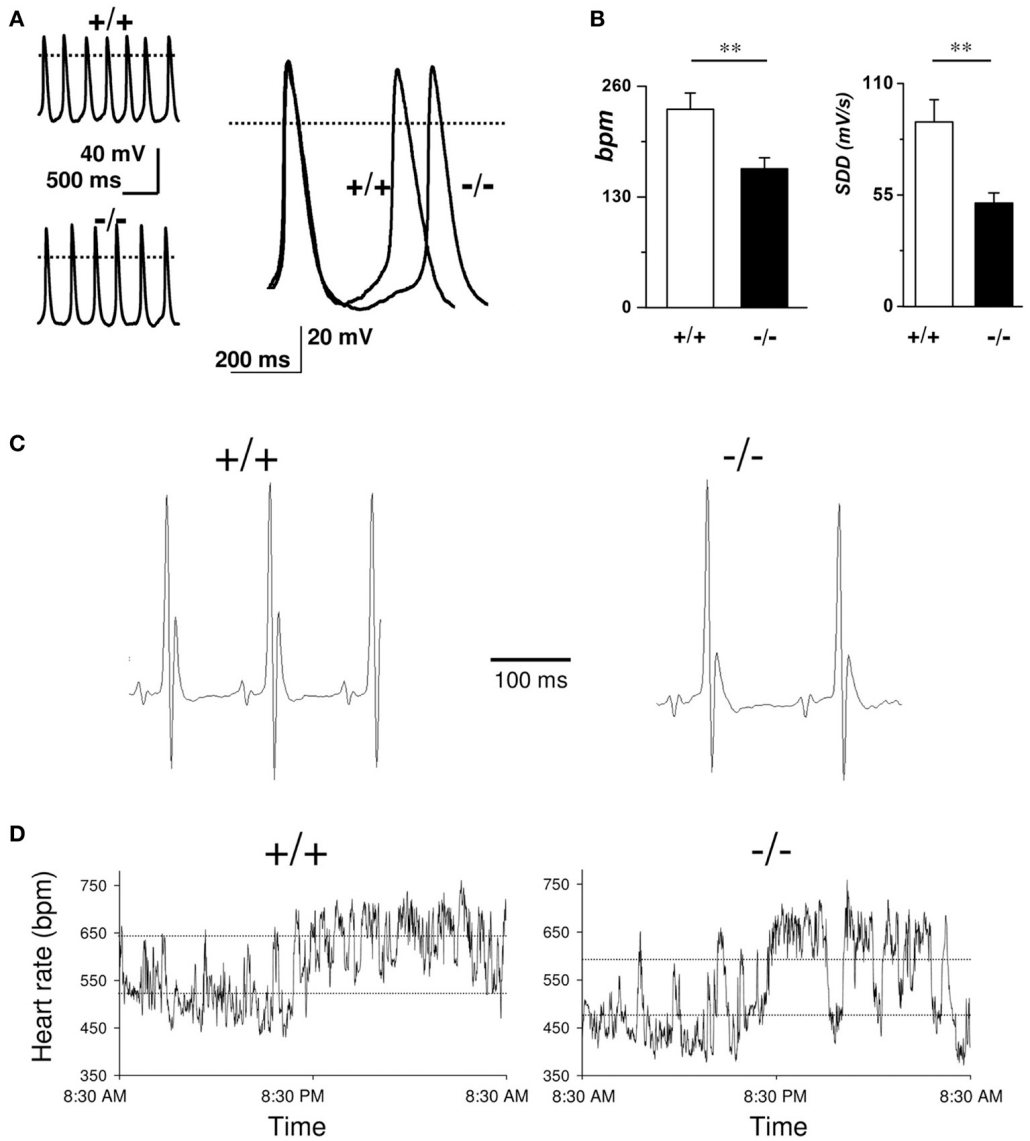
**Role of T-type VGCCs in cardiac automaticity**. **(A)** Representative sweeps of spontaneous action potentials obtained from SAN cells from WT (upper left trace) and Ca_v_3.1^−/−^ mice (lower left trace). Right panel: Superimposition of typical action potentials from a WT and from Ca_*v*_3.1^−/−^ SAN cell. **(B)** Histograms of the average bpm value and the slope of the diastolic depolarization (SDD). **(C)** Representative telemetric ECG recordings obtained on WT (left panel) and Ca_v_3.1^−/−^ animals (right panel). **(D)** Variation of heart rate (in bpm) in WT (left panel) and Ca_v_3.1^−/−^ mice (right panel) over a 24-h period. Dashed lines indicate mean day and night heart rates (reprinted from Mangoni et al., 2006b with permission from Wolters Kluwer Health).

In comparison to wild-type littermates mice lacking Ca_v_3.2 T-type channels do not show any significant differences in heart rate or the ECG waveform morphology; furthermore, no cardiac arrhythmias were observed in Ca_v_3.2 deficient mice indicating that Ca_v_3.2 mediated *I_Ca,T_* do not contribute significantly to the generation and the conduction of the cardiac impulse (Chen et al., 2003). Contrary to what reported for Ca_v_3.2 deficient mice, genetic inactivation of the Ca_v_3.1 T-type Ca^2+^ channels in mice results in a moderate bradycardia and significant slowing of AV conduction. Moreover, SAN and AVN cells of Ca_v_3.1^−/−^ hearts do not show residual Ca_v_3.2 mediated *I_Ca,T_*. Niwa et al. (2004) and Ferron et al. (2002) showed that the embryonic myocardium express Ca_v_3.2 channels, while the adult heart shows a higher expression of Ca_v_3.1 channels. These results suggest that Ca_v_3.2 underlies the functional T-type Ca^2+^ channels in the embryonic murine heart, and there is a subtype switching of transcripts from Ca_v_3.2 to Ca_v_3.1 in the perinatal period. As stated previously, ablation of Ca_v_3.1 subunits causes heart rate reduction (around 10%) and prolongation of the PQ interval due to first-degree atrioventricular block (Mangoni et al., 2006b). Similar results are obtained in sedated Ca_v_3.1^−/−^ mice after autonomic blockade by atropine and propranolol indicating a direct impact of Ca_v_3.1 subunits deletion in the SAN automaticity (Mangoni et al., 2006b) (Figure 3).

In agreement with this observation *in vivo*, pacemaker activity in isolated SAN cells is slowed by about 30% (Mangoni et al., 2006b). The relatively lower impact of *I_Ca,T_* loss of function on pacemaking *in vivo* compared to isolated cells can be explained supposing a shift of the leading pacemaker site in Ca_v_3.1^−/−^ SAN. This phenomenon is known as “pacemaker shift,” and can be observed when the SAN is challenged with neurotransmitters or pharmacologic agents that regulate pacemaker activity (Boyett et al., 2000; Lang et al., 2011; Inada et al., 2014). In the case of Ca_v_3.1^−/−^ hearts, it can be hypothesized that the leading pacemaker site of intact SAN is shifted to a location that is less sensitive to *I_Ca,T_* than the leading site in wild-type hearts. In this respect, pacemaker shift can be viewed as a compensatory mechanism to keep SAN rate as high as possible in the absence of Ca_v_3.1 channels. This hypothesis would need direct testing by employing optical or electrical mapping of pacemaking in wild-type and Ca_v_3.1^−/−^ SANs Similar hypothesis concerning pacemaker leading site shift have already been proposed to explain beat-to-beat variability, sinus node dysrhythmia and sinus pauses in mice lacking HCN1 channel (Fenske et al., 2013) or to partially explain the phenotype of human patients affected by “ankyrin B syndrome,” a disease characterized by sinus node dysfunction and increased susceptibility to spontaneous atrial fibrillation cause by Ankyrin-B dysfunction (Wolf et al., 2013). Another possible hypothesis to explain the difference in the pacing rate between isolated SAN cells and the heart rate *in vivo* in Ca_v_3.1^−/−^ mice would be the functional coupling between cardiac fibroblast and SAN myocytes. Indeed, it has been proposed that cardiac connective tissue facilitates impulse conduction *in vivo* (Camelliti et al., 2004; Kohl and Gourdie, 2014). Consequently, disruption of the electro-tonic coupling between fibroblast and Ca_v_3.1^−/−^ SAN cells during the cell isolation process could contribute reduce the pacing rate of isolated knockout myocytes. This hypothesis could also hold for other murine models lacking ion channels involved in pacemaker activity.

The prolongation of PQ interval in Ca_v_3.1^−/−^ mice suggested an important role of *I_Ca,T_* in AVN pacemaker cells. No residual *I_Ca,T_* was recorded in Ca_v_3.1^−/−^ AVN cells (Figure 2B) and the loss of Ca_v_3.1 mediated *I_Ca,T_* had remarkable effects on AVN cells automaticity. Pacemaker activity in Ca_v_3.1^−/−^ AVN isolated cells was irregular and slower (40%) than that of control cells (Marger et al., 2011a) suggesting that the relative importance of these channels in AVN automaticity may be even higher than that of SAN.

The importance of T-type channels in automaticity has been also investigated also in the ventricular conduction system. Le Quang et al. (2013) performed a clever study on the role of Ca_v_3.1 T- type Ca^2+^ channels subunits in escape rhythms and in bradycardia induced ventricular tachyarrhythmia after atrioventricular block. Adult male mice lacking Ca_v_3.1 alpha subunits after induction of complete atrioventricular block showed slower escape rhythms, greater mortality and higher frequency of torsades de pointes than control mice. This study suggests that Ca_v_3.1 channels play an important role in infra-nodal escape automaticity. Loss of Ca_v_3.1 channels also worsens bradycardia-related mortality, increases bradycardia-associated adverse remodeling, and enhances the risk of malignant ventricular tachyarrhythmia following atrioventricular block.

Although data from different studies show clearly the involvement of *I_Ca,T_* in cardiac automaticity and impulse conduction, mechanistic description of how T channels contribute to the diastolic depolarization is still lacking. Protas et al. (2001) proposed, for rabbit SAN cells, the existence of T-type window current component in the diastolic depolarization, but such a window current was not recorded either in the original study by Hagiwara et al. (1988) or Mangoni et al. (2006b) leaving this aspect still controversial. A previous study by our group employing numerical modeling of pacemaker activity of SAN and AVN mouse cells suggested that about 25 pA/pF of Ca_v_3.1-mediated *I_Ca,T_* flows during the diastolic depolarization (Hagiwara et al., 1988; Mangoni et al., 2006b; Marger et al., 2011a). Such a current density would be in theory sufficient to functionally contribute to the diastolic depolarization, since previous modeling work indicated that given the very high membrane resistance of SAN pacemaker cells at the maximum diastolic potential, a net inward current density as low as 1 pA/pF could initiate the diastolic depolarization (Difrancesco, 1993; Verheijck et al., 1999). Another hypothesis on how T-type channels can contribute to the pacemaking has emerged from the study by Huser et al. (2000). The paper states that in cat SAN and latent atrial pacemaker cells, the activation of T-type calcium channels during the late phase of the depolarization triggers elementary Ca^2+^ release events (Ca^2+^ sparks) from the SR which in turns stimulate NCX current to depolarize the pacemaker potential to threshold. These data were confirmed using 40 μM nickel (blocker of low voltage activated *I_Ca,T_*). Effectively, nickel reduced Ca^2+^ sparks and the slope of the diastolic depolarization, suggesting a functional coupling between T-type channels and SR (Lipsius et al., 2001), which could explain previous results showing that prevention of SR Ca^2+^ release with 10 μM ryanodine reduced T-type Ca^2+^ current (Li et al., 1997). However, Vinogradova et al. (2002) showed that nickel 50 μM slightly decreased the spontaneous cycle length of rabbit SAN cells and did not decrease the number of SR Ca^2+^ release suggesting a cell-type dependent role of *I_Ca,T_* in beating rate, SR Ca^2+^ release and diastolic depolarization. Therefore, the fact that *I_Ca,T_* appears to play a more important role in cat atrial latent pacemaker activity (Huser et al., 2000) than in primary pacemaker activity of rabbit SAN cells might be explained on the basis of a more negative maximum diastolic potential in atrial subsidiary vs. SAN cells (Vinogradova et al., 2002).

## Diseases of heart rhythm and cardiac VGCCs

During the last years, mutations in ion channels contributing to cardiac automaticity in humans have been described (Dobrzynski et al., 2007; Sanders et al., 2014). These mutations underlie complex arrhythmic profiles in affected patients. Typical clinical profiles include bradycardia due to sinus node dysfunction (Baig et al., 2011), atrioventricular block (Brucato et al., 2000) and ventricular tachycardia (Ueda et al., 2004). In particular, the discovery of two congenital pathologies of heart automaticity and atrioventricular conduction that could be attributed to a down regulation or loss-of-function of Ca_v_1.3 and/or Ca_v_3.1 channels highlights the physiological relevance of VGCCs in the determination of heart rate and atrioventricular conduction in humans. In this context genetically modified mice lacking Ca_v_1.3 or Ca_v_3.1 channels are important tools to test mechanistic hypothesis linking ion channel loss-of-function to bradycardia in affected subjects and for testing potential therapeutic strategies.

**Figure 4:**
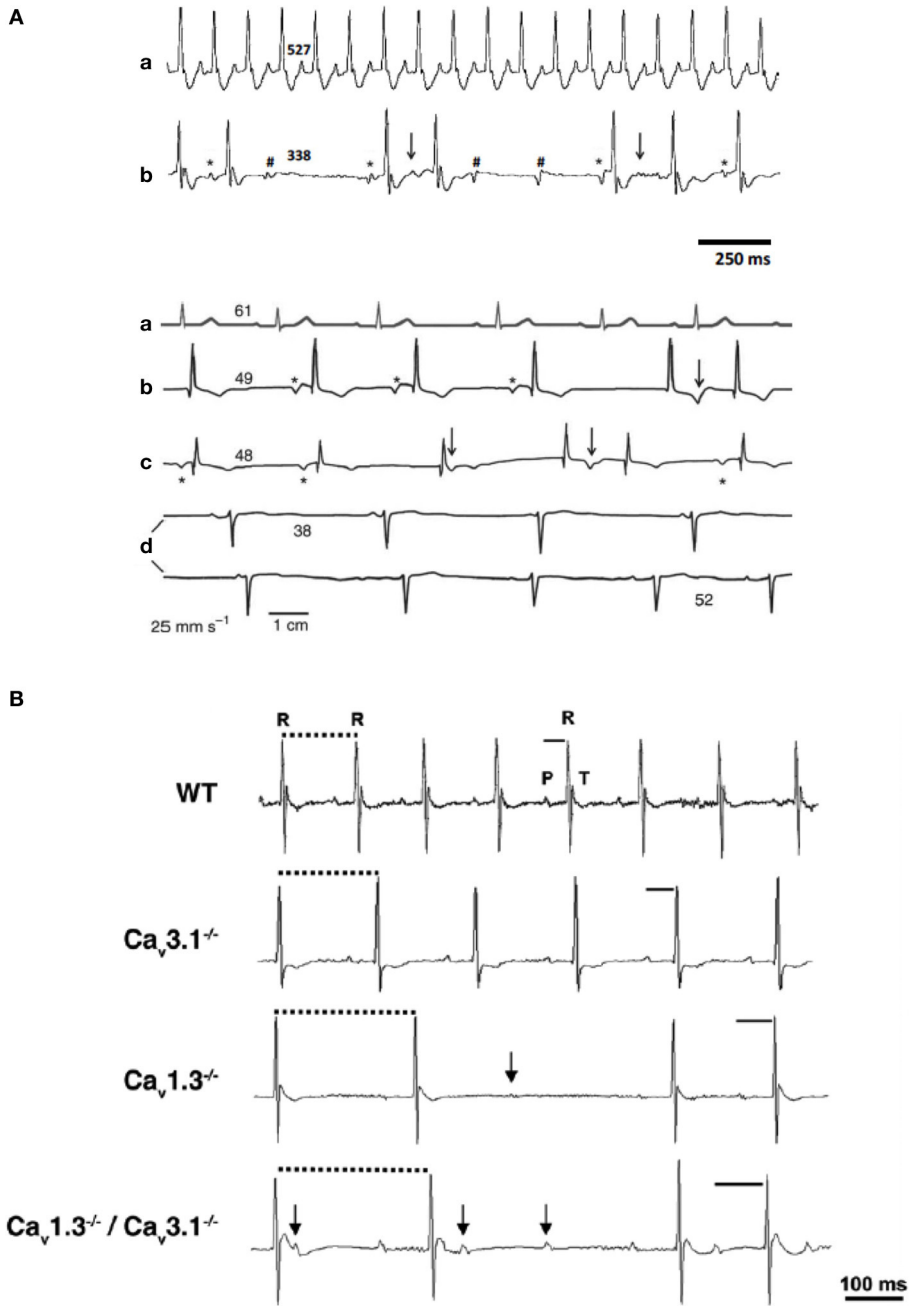
**Cardiac VGCCs in cardiac automaticity pathology**. **(A)** ECG sample recordings from WT (a) and Ca_v_1.3^−/−^ mice (b). **(B)** ECG recordings from a healthy person (a) and three individuals with SANDD syndrome (b–d). Asterisks mark P waves that precede QRS complexes; arrows indicate waveforms that suggest P waves coinciding with T waves; hashes indicate not conducted P waves. Numbers indicate heart rate (bpm) calculated from the corresponding beat-to-beat R-R interval (adapted from Baig et al., 2011). **(B)** Telemetric surface ECGs of freely moving WT, Ca_v_3.1^−/−^, Ca_v_1.3^−/−^, and Ca_v_1.3^−/−^/Ca_v_3.1^−/−^ mice showed additive effect of Ca_v_ gene inactivation on atrioventricular conduction dysfunction. Solid bars indicate PQ interval, dotted bars indicate RR intervals and arrows indicate isolated P waves (reprinted from Marger et al., 2011a with permission from Taylor and Francis LLC http://www.tandfonline.com).

Mice lacking Ca_v_1.3-mediated *I_Ca,L_* are phenotypically characterized by bradycardia and deafness (Platzer et al., 2000; Mangoni et al., 2003). Similar dysfunctions were discovered in two consanguineous families from Pakistan (Baig et al., 2011). Deep hearing loss and SAN dysfunction in these individuals closely are reminiscent of the phenotype of Ca_v_1.3^−/−^ mice. Because of the association between deafness and bradycardia, this newly described disease was named Sino-atrial Node Dysfunction and Deafness syndrome (SANDD). Patients with SANDD present SAN bradycardia at rest and exhibit variable degree of atrioventricular block and dissociated rhythms. This last observation can be explained by a recent result showing that Ca_v_1.3 is important for automaticity of mice AVN cells (Marger et al., 2011a) (Figure 4A). No short or long QT syndrome (LQTS) was reported in SANDD patients, indicating that Ca_v_1.3 channels do not directly participate to ventricular repolarization in humans. On the other hand recent data indicate that mutations in genes affecting regulation of Ca_v_1.2 channels can affect action potential duration. Particularly, mutations in calmodulin have been shown to be associated with cathecolaminergic polymorphic ventricular tachycardia (CPVT) and cardiac arrest (Nyegaard et al., 2012; Crotti et al., 2013). Limpitikul et al. (2014) showed that expression of mutated calmodulin suppressed Ca^2+^/calmodulin mediated CDI in native Ca_v_1.2 channels of ventricular myocytes. Suppression of CDI increased action potential durations and augmented the SR Ca^2+^ content. These works indicate that alteration in Ca_v_1.2 channels can induce LQTS (Limpitikul et al., 2014).

Recently, Neco et al. (2012), using a mouse model of CPVT carrying a mutation in RYR2 (RyR2^R4496C^), demonstrated a strong implication of SAN L-type channels in bradycardia associated with CPVT syndrome. RyR2^R4496C^ mice manifested alteration in Ca^2+^ homeostasis together with SAN dysrhythmia (SAN pauses) and impaired positive chronotropic response to β-adrenergic stimulation. Isolated RyR2^R4496C^ SAN cells showed Ca^2+^-dependent decrease of *I_Ca,L_* density, together with depletion of SR Ca^2+^ load during the diastolic phase, two factors that impaired the generation of SAN action potential. Ca^2+^ dependent inactivation by excessive RYR dependent Ca^2+^ release provides a new mechanistic rationale of SAN dysfunction in CPVT disease. It has been shown that not only inherited, but also acquired cardiomyopathy can involve L-type Ca_v_1.3. Rose et al. (2011) described a strong cardiac phenotype in a mouse model of chronic iron overload (CIO). SAN cells from CIO mice showed a strong decrease in Ca_v_1.3-mediated *I_Ca,L_* density. This decrease in *I_Ca,L_* induced bradycardia, sinus pauses, prolonged PQ intervals and second degree heart block *in vivo*.

Congenital heart block (CHB) disease is another pathology in which cardiac VGCCs are strongly implicated. CHB disease affects fetuses and newborns. CHB is an acquired autoimmune disease that occurs during pregnancies of rheumatic mothers, but also in healthy mothers. CHB is usually diagnosed between weeks 18 and 24 of pregnancy by fetal echocardiography techniques. Although it may initially appear as a first- or second-degree atrioventricular block, most cases present with fetal bradycardia and complete third-degree atrioventricular block. Other arrhythmias, including sinus bradycardia, diverse atrial rhythms, and junctional ectopic and ventricular tachycardia, have also been reported in the context of CHB (Ambrosi et al., 2014). While the etiology of this disease has remained obscure for long time, there is now strong evidence that loss-of-function of Ca_v_1.3 and Ca_v_3.1 channels underlie this pathology (Strandberg et al., 2013). Hu et al. (2004) have reported inhibition of *I_Ca,L_* and *I_Ca,T_* by immunoglobulin G isolated from mothers having CHB-affected children. SAN bradycardia and CHB can be explained at least in part by down regulation of Ca_v_1.3 and Ca_v_3.1 channels by maternal antibodies (Hu et al., 2004) suggesting a strict correlation between loss of function of Ca_v_1.3 and Ca_v_3.1 VGCCs and CHB. Results published by our group (Marger et al., 2011a) further support this hypothesis. Indeed, we studied heart rate and atrioventricular conduction in mice with combined inactivation of Ca_v_1.3 and Ca_v_3.1 channels (Ca_v_1.3^−/−^/Ca_v_3.1^−/−^) showing that Ca_v_3.1 and Ca_v_1.3 inactivation have an additive effect on atrioventricular conduction (Figure 4B). Indeed, while inactivation of Ca_v_3.1 channels alone causes moderate dysfunction of atrioventricular conduction, association with Ca_v_1.3 inactivation induces severe atrioventricular block. Some Ca_v_1.3^−/−^/Ca_v_3.1^−/−^ mice display complete block with dissociated atrial and ventricular. Disruption of both Ca_v_1.3 and Ca_v_3.1 subunits has also additive effects on AVN cells pacemaking. Ca_v_1.3^−/−^/Ca_v_3.1^−/−^ AVN cells display poor or absent automaticity, thus stressing the importance of voltage-dependent Ca^2+^ channels in pacemaker activity of these cells. These results indicate that Ca_v_1.3^−/−^/Ca_v_3.1^−/−^ mice constitute a faithful animal model of CHB and could be used for testing of new therapies (Marger et al., 2011a).

In conclusion, work on mouse models of SANDD and CIO (Ca_v_1.3 channels), CPVT (*I_Ca,L_*), and CHB (*I_Ca,L_* and *I_Ca,T_*) demonstrates that despite the differences between mouse and human cardiac rhythm the mouse is a valuable model for studying the role of ion channels in human pathologies of heart rhythm.

## Concluding remarks

The relevance of VGCCs in the generation and regulation of cardiac pacemaking, atrioventricular conduction and heart rate determination is now well established. Importantly, the functional role of VGCCs such as Ca_v_1.3 and Ca_v_3.1 channels seems to be conserved between rodents and humans. *I_Ca,L_* and *I_Ca,T_* play a major role in atrioventricular conduction as underscored by the presence of dissociated rhythms in Ca_v_1.3^−/−^ mice and SANDD patients (Baig et al., 2011) or in Ca_v_1.3^−/−^/Ca_v_3.1^−/−^ mice and CHB patients (Marger et al., 2011a). Future studies will further address the role of VGCCs in pacemaker activity and in particular their importance in respect to other ion channels involved in automaticity such as HCN4 and RYRs.

### Conflict of interest statement

The authors declare that the research was conducted in the absence of any commercial or financial relationships that could be construed as a potential conflict of interest.
